# R0 resection of linitis plastica of the stomach with synchronous bilateral Krukenberg tumours in a young woman

**DOI:** 10.1093/jscr/rjae053

**Published:** 2024-02-23

**Authors:** Venkiteswaran Muralidhar, Pooja E Moorthy, Akshay C K Krishnan, Leo J Manavalan

**Affiliations:** Department of Surgery, Chettinad Academy of Research and Education, Kelambakkam, Chennai 600103, India; Department of Pathology, Chettinad Academy of Research and Education, Kelambakkam, Chennai 600103, India; Department of Surgery, Chettinad Academy of Research and Education, Kelambakkam, Chennai 600103, India; Department of Surgery, Chettinad Academy of Research and Education, Kelambakkam, Chennai 600103, India

## Abstract

We report a case of linitis plastica (LP) with synchronous bilateral Krukenberg Tumours in a young woman, which could be resected fully. Such a case is rarely reported because of rarity (LP), dismal prognosis (LP and Krukenberg Tumours), nonresectability due to peritoneal spread at presentation, and lack of clear treatment protocols (LP and Krukenberg Tumours). This case report suggests that LP, with Krukenberg Tumours, can achieve complete resection in a select subset of cases; this may improve survival.

## Introduction

LP is a macroscopic infiltration of the gastric wall with diffuse signet ring histopathology [1]. Resection with curative intent (R0) in linitis plastica (LP) with synchronous Krukenberg Tumours (KT) is rarely reported [1]. LPs are rare tumours and peritoneal spread precludes R0 resection [2]. Gastric cancer is the leading cause of KT in women [3]. The treatment protocol for KT after curative gastric resection is unclear [4, 5]. Studies have shown that a complete resection (R0) of LP, if possible, improves survival [2, 6, 7]. Furthermore, surgical resection of metastasis has been shown to improve survival after curative gastrectomy for all types of gastric cancer, including LP [4]. We report a case of LP in a young woman who had synchronous bilateral KTs, which was amenable to surgical resection of all macroscopic tumours (R0).

## Case report

**Figure 1:**
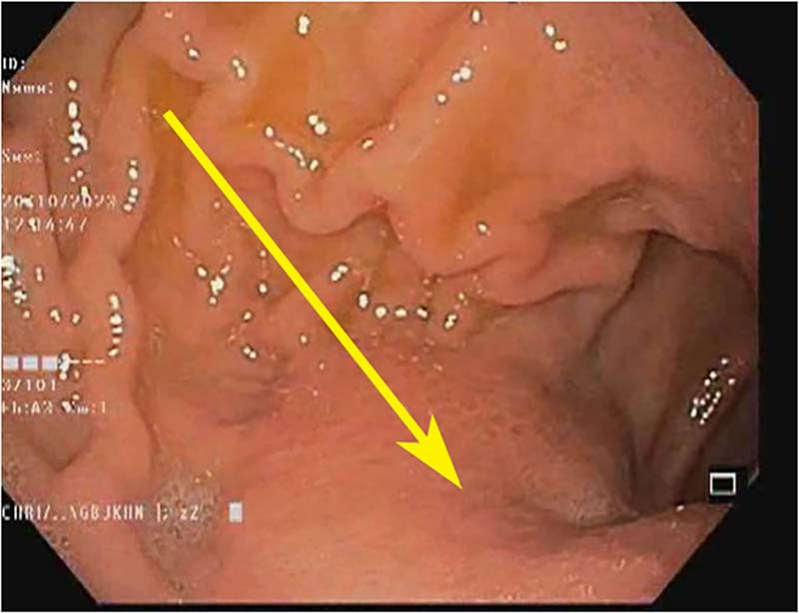
Endoscopic image showing the gastric antrum: loss of rugosity, erythema, and extramucosal bulge (arrow).

**Figure 2:**
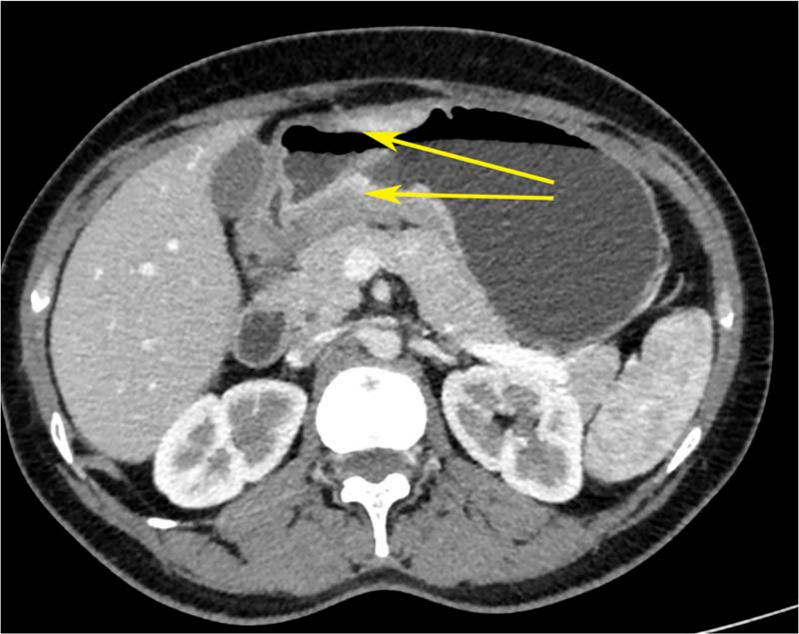
CT scan of the abdomen showing gastric wall thickening at the level of the pyloric antrum (yellow arrows).

**Figure 3:**
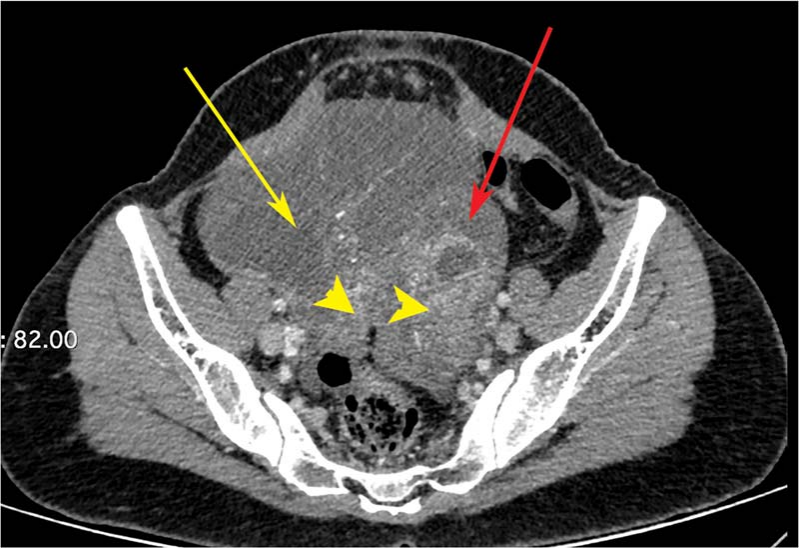
CT scan of the abdomen showing right ovarian cyst (yellow arrow), left ovarian cyst (red arrow) with solid elements within (arrow heads).

A woman in her early 30s, with no prior morbidities, presented with fullness in the epigastrium and a weight loss of 5 kg in the past month. She had a regular menstrual history and a history of two full-term normal deliveries. Her last child was born 5 years ago. She had no relevant medical history. On examination, her pulse was 120 beats per minute, blood pressure was 160/80 mm of mercury, respiratory rate was 20 per minute, and Glasgow coma scale was 15. Hernial orifices were normal. Abdominal and chest examinations were normal. There was no lymphadenopathy. Examinations of skin, skull, and spine were normal. There was nothing significant on per rectal and per vaginal examination. A PA X-ray of the chest showed normal findings. Blood investigations showed: haemoglobin: 10.5 g/dl, total leucocyte: 7.4 × 100/l, serum creatinine: 0.72 mg/dl, random blood sugar: 86 g/dl, alanine transaminase: 24 U/l, aspartate transaminase: 16 U/l, alkaline phosphatase: 35 U/l, and total bilirubin: 0.8 mg/dl. Esophagogastroduodenoscopy was performed, which showed erythema, loss of rugosity and an extra mucosal bulge at the antrum; mucosal biopsies were positive for malignancy (Fig. 1). Tumour markers CA-125 and CA-19-9 were normal. The distal stomach did not expand with insufflation, suggesting diffuse infiltration. Serology testing of *Helicobacter pylori* was negative. An abdominal CT scan showed a bilateral ovarian cyst with solid components and gastric antral wall thickening (Figs 2 and 3). There was no lymphadenopathy, liver spleen, urinary bladder kidney lesions, or ascites. The diagnosis was LP of the stomach with bilateral Krukenberg tumours (KT). A diagnostic laparoscopy showed no peritoneal disease on multiple biopsies and washes. It also showed no gastric serosal infiltration, and it was possible to get an R0 resection.

**Figure 4:**
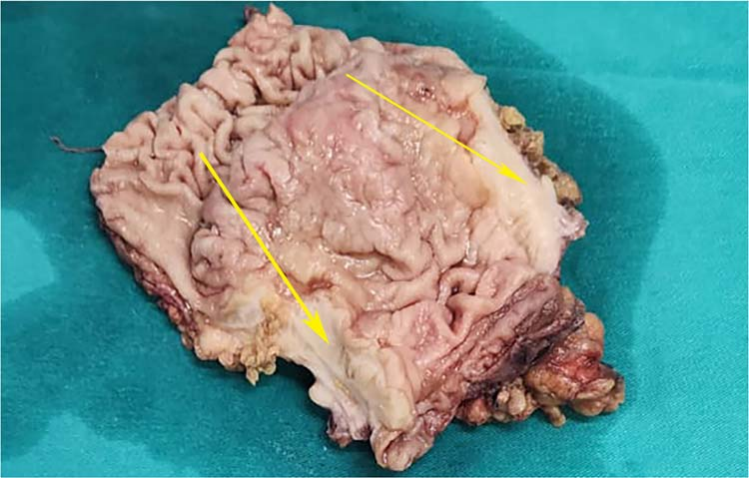
Postoperative specimen of the stomach showing intramural tumour at the pyloric antrum (arrow).

**Figure 5:**
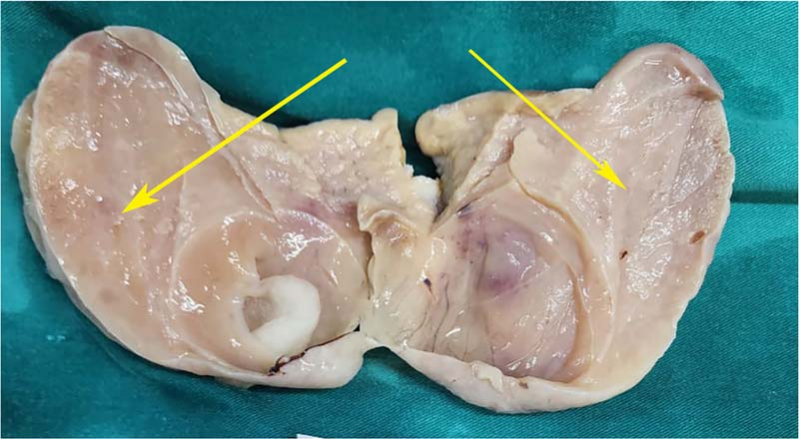
Postoperative specimen of the right ovarian cyst showing solid areas within (arrows).

**Figure 6:**
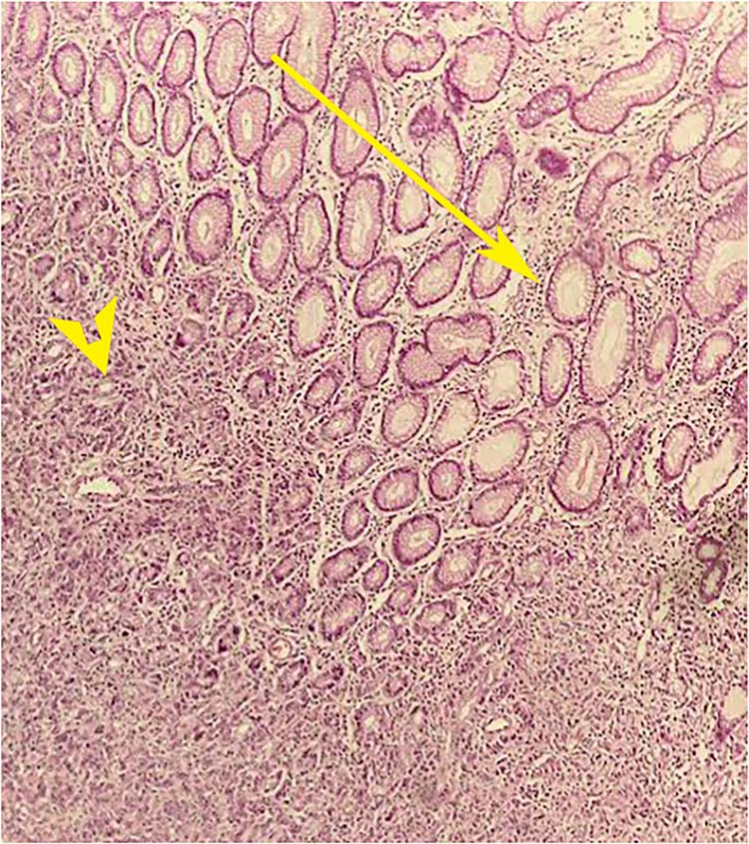
Postoperative histopathology H&E stain: 10× magnification showing tumour (arrow head) in the gastric wall pushing into the intact mucosa (arrow).

**Figure 7:**
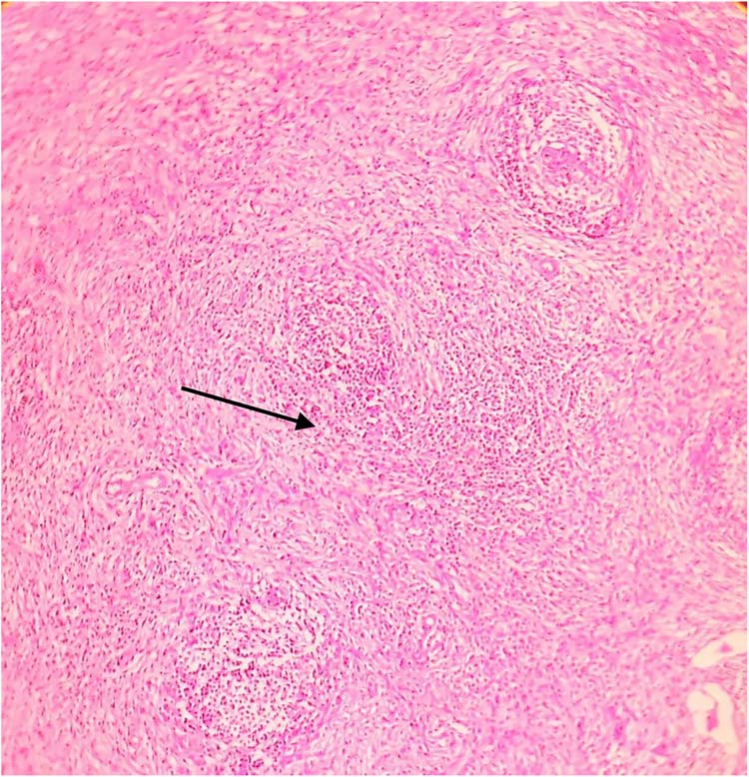
Postoperative histopathology H&E stain: 40× magnification of tumour in the gastric wall showing signet cells and desmoplastic reaction.

**Figure 8:**
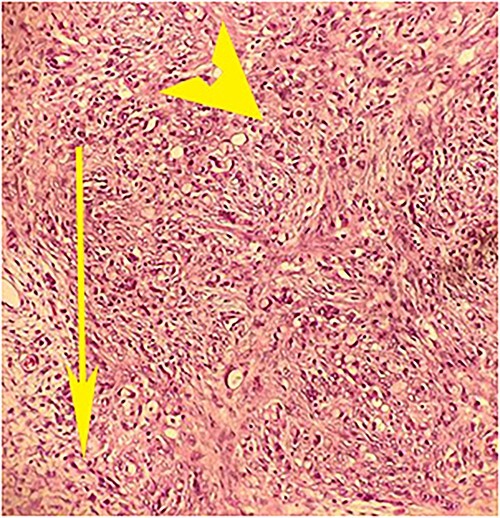
Postoperative histopathology H&E stain: 20× magnification of ovarian tissue showing tumour with signet cells (arrow head) and oedematous surrounding stroma (arrow).

**Figure 9:**
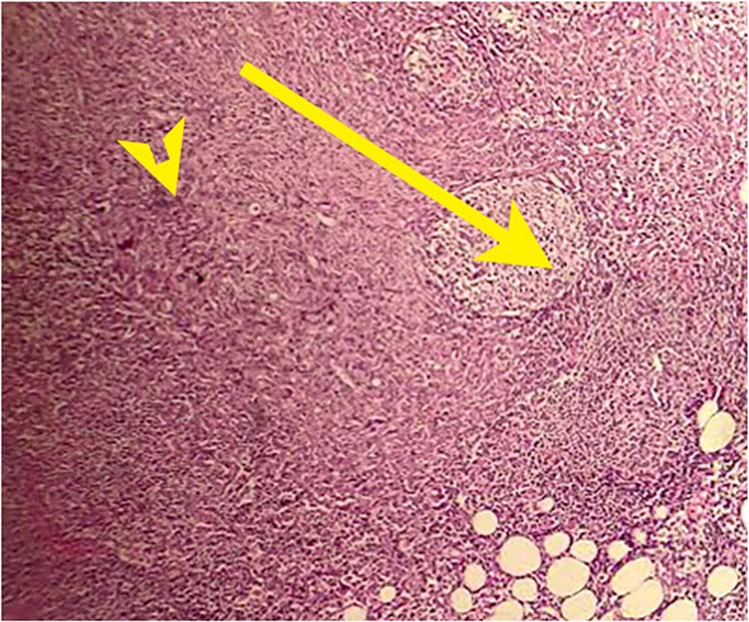
Postoperative histopathology H&E stain: 10× magnification of a representative perigastric lymph node showing tumour (arrow head) surrounding the germinal centres (arrow).

On laparotomy, the gastric antrum was infiltrated with a tumour with no serosal extension (Fig. 4), no ascites, and bilateral ovarian cysts (right: 12 × 10 × 6 cm, left: 9 × 5 × 4 cm) (Fig. 5). An R0 resection that included D2 gastrectomy and pan-hysterectomy was performed. The patient made an uneventful recovery and was discharged on the 15th postoperative day. The final diagnosis after histopathological examination was diffuse infiltrating gastric carcinoma signet cell-type (Figs 6 and 7), LP, KT deposits in both the ovaries (Fig. 8), with regional lymph node involvement (Fig. 9) in two nodes among the 15 nodes that were dissected. The staging was T4a, N1, and M1. The patient decided against adjuvant treatment. She was disease-free on clinical and radiological examinations at 12 months.

## Discussion

LP is a rare tumour associated with a dismal prognosis with a 5-year survival of <10% [2, 8, 9]. The optimal management of LP is controversial [1]. Surgical resection of LP significantly improves survival [2, 6, 7]. Chemotherapy does not improve survival in LP [1, 10, 11]. Studies have shown a clear survival benefit of resection of KT after curative gastric resection for gastric cancers, including LP [4, 12, 13]. Survival benefits after resection of LP are possible only in a small subset of cases that do not have peritoneal spread [2]. A retrospective study has shown that, among 94 patients of gastric cancer with synchronous KT, metastasectomy improves overall survival by eight months (*P* = .001) [4]. There were 10 cases of KT in the study who fared worse than non-LT gastric cancer patients who underwent metastasectomy, but the difference was not significant (*P* = .020) [4]. To summarize, KT with ovarian metastasis, a rare occurrence, can be treated with R0 resection in a select subset of women, which may improve survival.
