# Correction to: R0 resection of linitis plastica of the stomach with synchronous bilateral Krukenberg tumours in a young woman

**DOI:** 10.1093/jscr/rjaf561

**Published:** 2025-09-25

This is a correction to: Venkiteswaran Muralidhar, Pooja E Moorthy, Akshay C K Krishnan, Leo J Manavalan, R0 resection of linitis plastica of the stomach with synchronous bilateral Krukenberg tumours in a young woman, *Journal of Surgical Case Reports*, Volume 2024, Issue 2, February 2024, rjae053, 10.1093/jscr/rjae053

In the originally published version of this manuscript, the following affiliation was mistakenly omitted for author Venkiteswaran Muralidhar:

University of Birmingham, Birmingham B15 2TT, United Kingdom of Great Britain and Northern Ireland.

These details have been corrected only in this correction notice to preserve the published version of record.

